# Uncommon life history and winter spawning of common carp (*Cyprinus carpio*) in a natural thermal spring, under temperate climate

**DOI:** 10.1007/s10695-024-01305-w

**Published:** 2024-01-30

**Authors:** Tamás Müller, Árpád Ferincz, András Weiperth, Bence Ivánovics, Balázs Tóth, Bence Bógó, József Horváth, Béla Urbányi, András Specziár

**Affiliations:** 1 Department of Freshwater Fish Ecology, Institute of Aquaculture and Environmental Safety, Hungarian University of Agriculture and Life Sciences, 7400 Gödöllő, Hungary; 2 Department of Systematic Zoology and Ecology, Institute of Biology, Eötvös Loránd University, 1117 Budapest, Hungary; 3 Department of Environmental Toxicology, Institute of Aquaculture and Environmental Safety, Hungarian University of Agriculture and Life Sciences, 2100 Gödöllő, Hungary; 4 Duna-Ipoly National Park Directorate, 2509 Esztergom, Hungary; 5 Department of Aquaculture, Institute of Aquaculture and Environmental Safety, Hungarian University of Agriculture and Life Sciences, 2100 Gödöllő, Hungary; 6 HUN-REN Balaton Limnological Research Institute, 8237 Tihany, Hungary

**Keywords:** Maturity, Breeding season, Dwarfism, Extreme habitat, Thermal habitat, Climate change

## Abstract

Common carp female generally matures at age 4–5 years old and spawns between April and July under the temperate climate. Contrary to a range of 0–28 °C of temperate freshwaters, the water temperature of Lake Hévíz (Hungary, Central Europe), the largest natural bathable thermal lake in the world, varies between 26 and 35 °C seasonally. The specific environmental conditions (continuously warm water and its individual chemical composition, special nutrient base, lack of natural lakeside spawning substrate compared to usual spawning grounds, continuous high human disturbance, etc.) suggest that the carp population here may also differ in reproductive characteristics from their counterparts in surrounding waters. Our findings suggest that the self-sustaining dwarf common carp population of Lake Hévíz matures 2 to 4 years earlier (at the age of one) and spawns 1 to 3 months before (between February and April, at 27–30 °C water temperature) than carp typically do in the temperate zone (16–20 °C). Successful winter spawning was verified by rearing larvae from the collected eggs and in situ induced propagation.

## Introduction

In Europe, common carp (*Cyprinus carpio*) has been under domestication since the Middle Ages. The cultivated varieties are believed to have originated from the wild populations found along the Danube River. These wild populations are naturally found in rivers that flow into the Black, Caspian, and Aral Seas. While common carp is extensively cultivated across the globe, it is worth noting that numerous cultivated varieties, especially those from Asia, actually stem from various other East Asian species (Freyhof and Kottelat 2008). Common carp is the fourth most important cultured fish species in aquaculture, with production reaching 4,411,900 metric tons in 2019 (FAO Fisheries Statistics).

There is a special dwarf wild common carp population living in Lake Hévíz (Hungary), which is the biggest, biologically active natural thermal lake in the world. The 4.44 ha water surface lake is surrounded by a 60.5 ha nature conservation area. The lake is fed by several thermal springs flushing into the central cave at 38 m depth and with temperatures ranging between 23.4 and 41.8 °C. Total discharge of 410 L s^−1^ of springs results a rapid replacement of the lake water with an average resident time of just 3 and half days. Water temperature of the lake varies between 33 and 35 °C in summer, and it does not decrease below 26–29 °C even during the coldest winters. High water temperature and individual chemical composition of Lake Hévíz resulted in the formation of a unique biological community (Ponyi 2002), including a special fish assemblage as well (Bíró et al. 2002; Specziár 2004). Food resource of the lake is very scarce (Ponyi 2002), and thus fish should feed on detritus to an unusual degree (Specziár 2004). Currently, only a few fish species have permanent populations; these are the exotic eastern mosquitofish (*Gambusia holbrooki*), the rainbow cichlid (*Archocentrus multispinosus*), the pumpkinseed sunfish (*Lepomis gibbosus*), and jewel cichlid (*Hemichromis guttatus*) (Lőkkös et al. 2016), and the only native fish is the special dwarf from of the wild common carp, which was described by Hermann (1888) from some thermal springs in Hungary. At the present, the only remained stock of this dwarf common carp is in Lake Hévíz. Pilot studies suggest that this population has very uncommon environmental tolerance and ecology (Herman 1887; Varga et al. 2013; 2021; Molnár et al. 2019). This population is isolated, self-sustaining, and stable. It consists of dwarf individuals up to 7+ years old but rarely exceeding 25 cm standard length and 500 g body weight. Note that 7+ age groups in nearby populations are often larger than 60 cm and 10 kg (Specziár 2010, Table 1). All fish over 12–17 cm and at 1 to 2 years old are matured and do not display any evident signs of malnutrition or disease. Preliminary observations on feeding, lipid reserves, and parasitological condition of this fish (Varga et al. 2013, 2021; Molnár et al. 2019) all suggest adaptation to this extreme habitat.

**Table 1:** Comparative data on common carp growth in Lake Balaton and its catchment area

	Lake Hévíz, *n* = 75, sampled in 2007–2010	Lake Balaton, sampled in 1996 (Specziár 2010)	Kis-Balaton wetland area, sampled in 1994–1996 (Tölg et al. 1996)
Age (year)	Standard length (mm)		
0+	83.3	85	105
1+	122.6	191	197
2+	151.6	289	280
3+	173.7	377	355
4+	191.9	457	422
5+	205.6	531	482
6+	208.8	597	536
7+	221.5	658	585

In temperate climate zone, the mature wild common carp in natural water systems spawns from the end of April to the end of June or sometimes the beginning of July. Spawning commences when the water temperature has reached 16 to 18 °C. In the Danube Basin and watersheds of other larger central and eastern European rivers, the wild carp typically spawns during periods of high water level on freshly flooded meadows, at water depth ranging between 25 and 50 cm and in the littoral zone where aquatic or terrestrial plants are represented (Barus et al. 2002). The specific environmental conditions (continuously warm water and its individual chemical composition, lack of cold water resting season and natural lakeside spawning substrate compared to normal spawning grounds, continuous high human disturbance, etc.) suggest that the common carp population here may also differ in reproductive characteristics from their counterparts in surrounding waters, and in general, in the temperate zone. Studying common carp populations in such special habitats improves our understanding on the adaptation capability of common carp to extreme environment and can provide important information about the probable impact of global climate change.

The aim of the study was to reveal the uncommon reproduction strategy of common carp in Lake Hévíz. Specifically, we attempted obtaining information about the reproductive characteristics of dwarf common carp, in particular, about the time of maturity, spawning period, and spawning habitat. We also attempted rearing of fertilised eggs collected from the natural spawning to larval stage in the laboratory and proving their viability and in situ induced propagation.

## Material and methods

### Spawning observation, spawning conditions, egg collections, GSI, and incubation

**Fig. 1:**
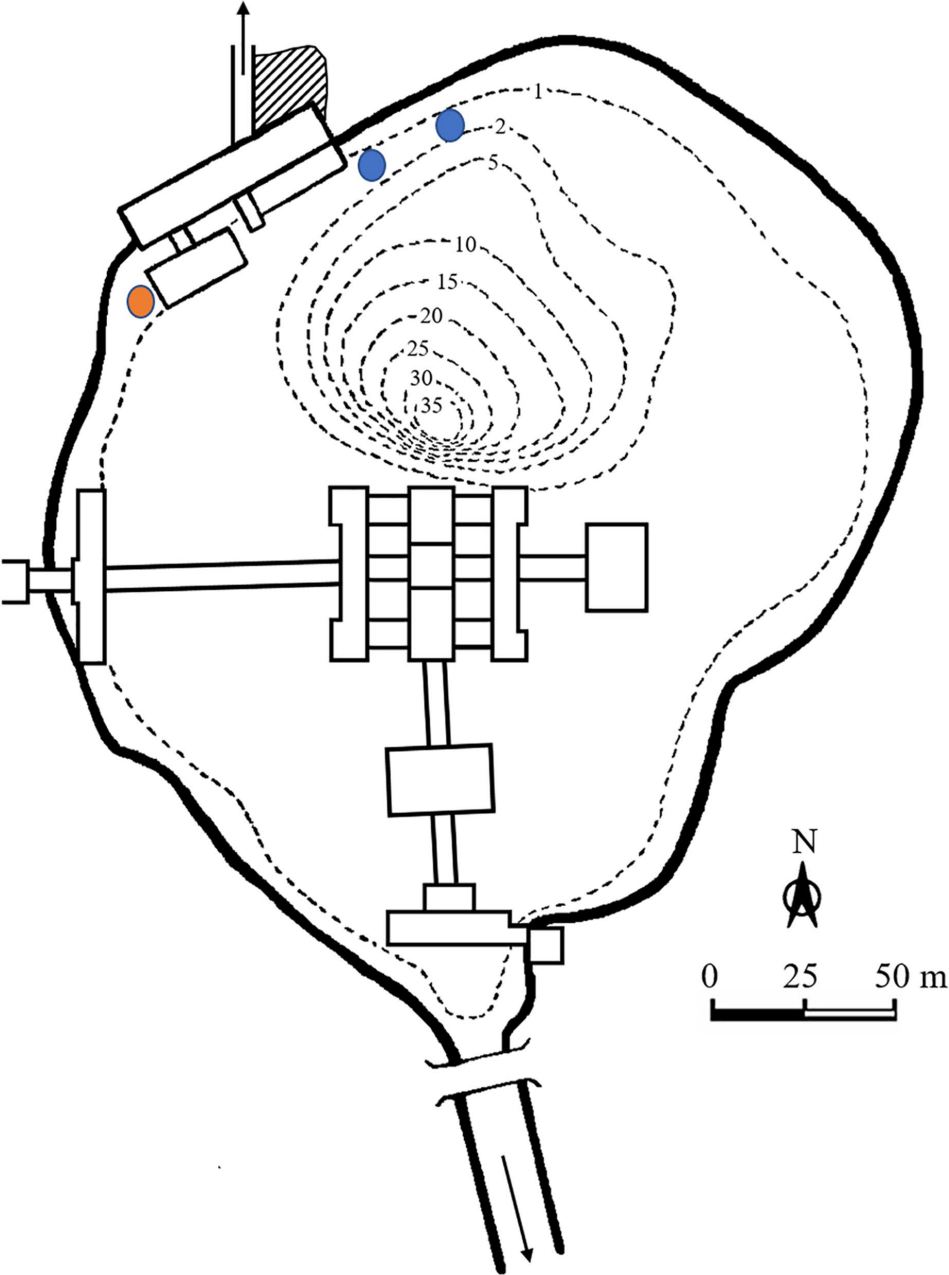
Lake Hévíz blank map. Dashed lines indicate bathymetric contours in m. Blue circle: observed spawning and egg collection, red circle: egg collection

The lake has no typical littoral zone as the shoreline is fixed with palisade (Fig. 1). The water parameters at the spawning site are given in Table 2. Natural spawning of dwarf common carp was first observed on 23 February 2021 in Lake Hévíz. Eggs from this spawning were collected and transported to Fish Laboratory of Department of Freshwater Ecology in Gödöllő, MATE. Embryos were incubated in the laboratory in original lake water at 26 °C. Images of embryos and larvae were taken by Leica M205 FA fully motorised fluorescence stereo microscope (Leica DFC 7000 T camera, Leica Application Suite X software, Leica Microsystems GmbH; Wetzlar, Germany). Egg diameter and total body length were measured by ImageJ software (data in Table 1). The gonadosomatic index (GSI) was calculated as an indicator of ovarian development in a total of 142 individuals sampled between 2007 and 2022: GSI (%) = [weight of gonad (g)/total body weight (g)] × 100.

**Table 2:** Physicochemical properties of water of Lake Hévíz at the time of observed carp spawning

Water parameter	Values	Measuring instrument
Water temperature	26.9 °C	Hanna HI 98194
pH	7.06	
Dissolved oxygen	2.98 mg L^−1^, 25.5%	
Conductivity	777 µS cm^-1^	
Total dissolved salinity in solution	388 ppm	
Chlorophyll-a	0.4 µg L^−1^	AlgaeTorch2 spectrofluorometer, bbe Moldaenke
Cyanobacteria	0.1 µg L^−1^	
[PO_4_]^3−^	< 0.2 mg L^−1^	Macherey-Nagel VisoColor PF12 spectrophotometer
[NO_3_]^−^	< 0.1 mg L^−1^	
[NO_2_]^−^	< 0.01 mg L^−1^	
[NH_4_]^+^	0.3 mg L^−1^	

### In situ induced propagation

Broodstocks were collected by representative gillnet sampling (European Standard, EN 14757:2005 multi mesh gillnets composed of 12 different mesh sizes ranging from 5 to 55 mm) with short checking time (≤ 30 min) to ensure the survival of captured fish. Females and males were selected based on conditional characteristics for propagation experiment. Before handling, fish were anaesthetised with clove oil in a plastic tank (20 drops 10 L^−1^). All fish were injected intraperitoneally with carp pituitary extract (5 mg per kg bodyweight) at 19:00–21:00 h. Detailed data about the experiments (Experiments I.–III.) have been presented in Table 3. Fish were introduced into a cage (height 500 mm, diameter 450 mm, volume 79 L) which bottom was covered with a plastic mesh (mesh size 0.75 mm; FYLLEN Laundry Basket©, IKEA IT AB, Sweden) serving as substrate for induced spawning.

**Table 3:** Experimental design for induced propagation

Experiment No.	Date (water temperature, °C)	Broodstock/spawning cage	Body weight (g)	Type of propagation and description of the experiment
Experiment I.	11.02.2019 (24.7 °C)	Female (*n* = 2)	70–125	*In vitro* fertilisation: Fish eggs and sperm were collected from freshly captured and anaesthetised individuals, and the fertilisation of the eggs occurred in petri dish at the lakeshore
		Male (*n* = 2)	82–123	
Experiment II.	22.02.2021 (26.9 °C)	Female (*n* = 2)	176–184	Induced spawning: The hormonally induced maturation process and the actual spawning of freshly captured fish happened in the cage placed into Lake Hévíz
		Male (*n* = 2)	128–145	
Experiment III.	09.03.2022 (25.3 °C)	Female (*n* = 1)	192	
		Male (*n* = 2)	110–138	

## Results

### Spawning observation, spawning conditions, egg collections, and incubation

**Fig. 2:**
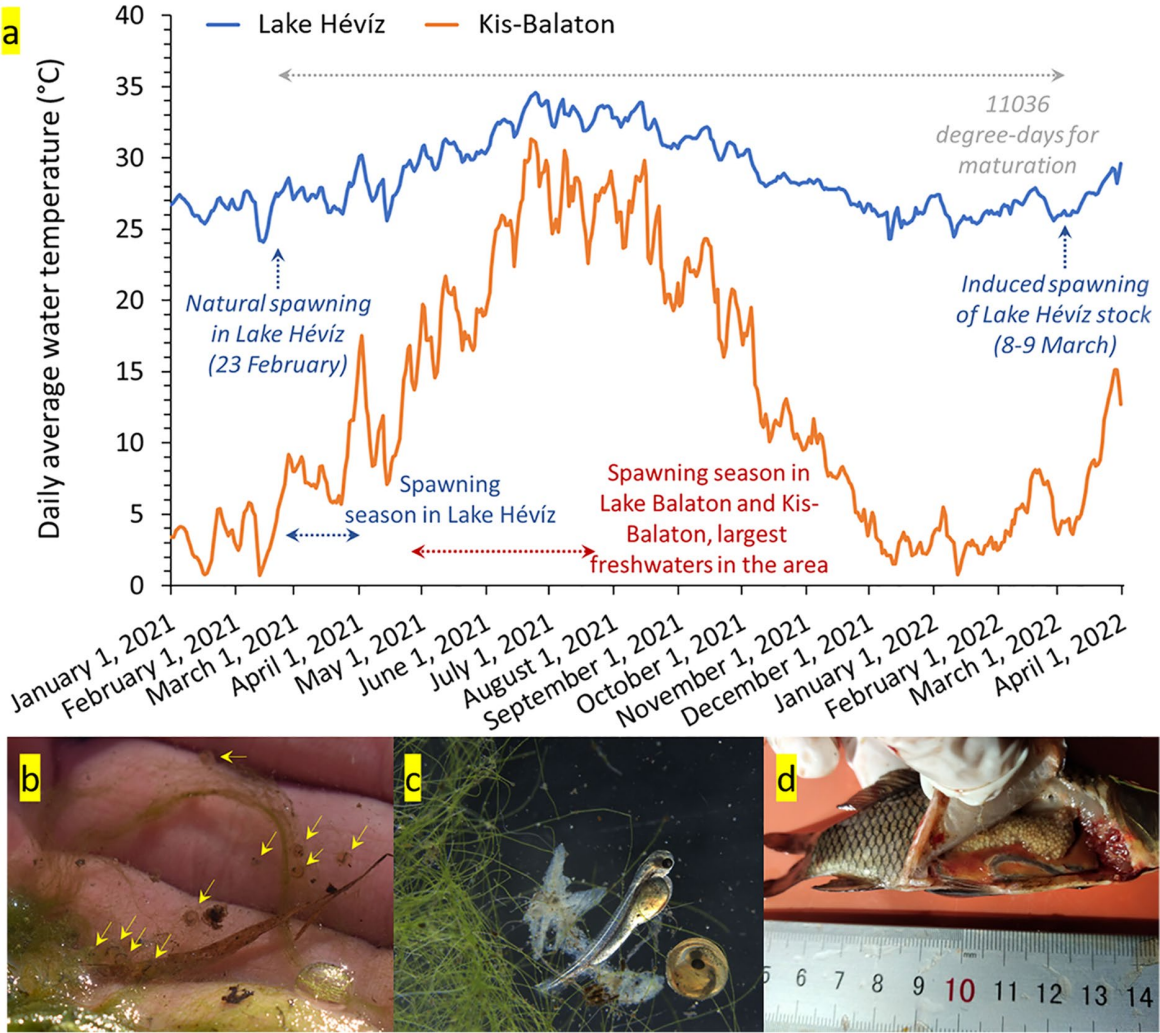
Summarised information about maturation and spawning conditions of wild dwarf common carp inhabiting Lake Hévíz. **a** Daily water temperature changes between January 2021 and April 2022 in Lake Hévíz and Kis-Balaton (Balatonmagyaród), the closest major freshwater habitat in the region, with indication of time period covering the required sum of degree-days to maturation (based on Horváth et al. 1977), natural spawning season, and date of a successful induced spawning. **b** *Cladophora* filaments with carp eggs collected in Lake Hévíz at 23 February 2021. Yellow arrows—eggs with eye stage embryos. **c** Embryo and freshly hatched carp larva from eggs collected in Lake Hévíz at 23 February 2021 and incubated in the laboratory. **d** The smallest mature female carp from Lake Hévíz (01 March 2022; body mass, 24 g; standard length, 90 mm; GSI, 7.09%; oocyte diameter, 886 ± 66 µm)

At areas of 1.7–2 m water depth, small groups of dwarf common carps (a female with 1–3 males) visibly spawned periodically among the introduced, long-flowered subspecies of Indian red water lily (*Nymphaea rubra* var. *longiflora*), spreading over the water surface between about 06:00 and 09:30 h. The spawning substrate was the under part of the leaves of the Indian red water lily colonised by filamentous green algae (*Cladophora* sp.). After the spawning, fish eggs were found on the *Cladophora* covering the shoreline-protecting palisade as well (Fig. 2). Common carp larvae were successfully hatched from the eggs collected. The sizes of the egg-containing embryos and freshly hatched larvae are shown in Table 4.

**Table 4:** Summarised reproduction characteristics of common carp for the temperate zone and Lake Hévíz

	Temperate zone (Horváth et al. 2002, Barus et al. 2002)	Lake Hévíz (period 2007–2022)
Age of maturity, males (year)	2–3	< 1
Age of maturity, females (year)	4–5	1
Natural spawning		
Spawning period	From end of April to end of July	From end of February to early April
Spawning temperature	16–20 °C	25–30 °C (26.9 °C*)
Dissolved oxygen at spawning site	5–6 mg L^−1^	2.98 mg L^−1^*
Spawning area and substrate	Freshly flooded meadows, shallow water (25–50 cm), areas covered with fine aquatic or flooded riparian vegetation	Deep water (up to 1.7 m), under side of leaves of Indian red water lily and filamentous algae (*Clapophora* spp.) covering the water lily and the shoreline stabilising palisade*
Other key environmental factors that trigger/facilitate spawning	Flooding, when the ionic content of the water is diluted, possibly due to the arrival of a warm front, which also causes a change in air pressure	Longer days
Swollen egg diameter (mm)	1.69–2.5	1.66 ± 0.14, *n* = 39* (min.–max., 1.40–1.98)
Total length of freshly hatched larvae (mm)	4.8–6.7	6.03 ± 0.5, *n* = 4* (min.–max., 5.63–6.39)

*Present study

**Fig. 3:**
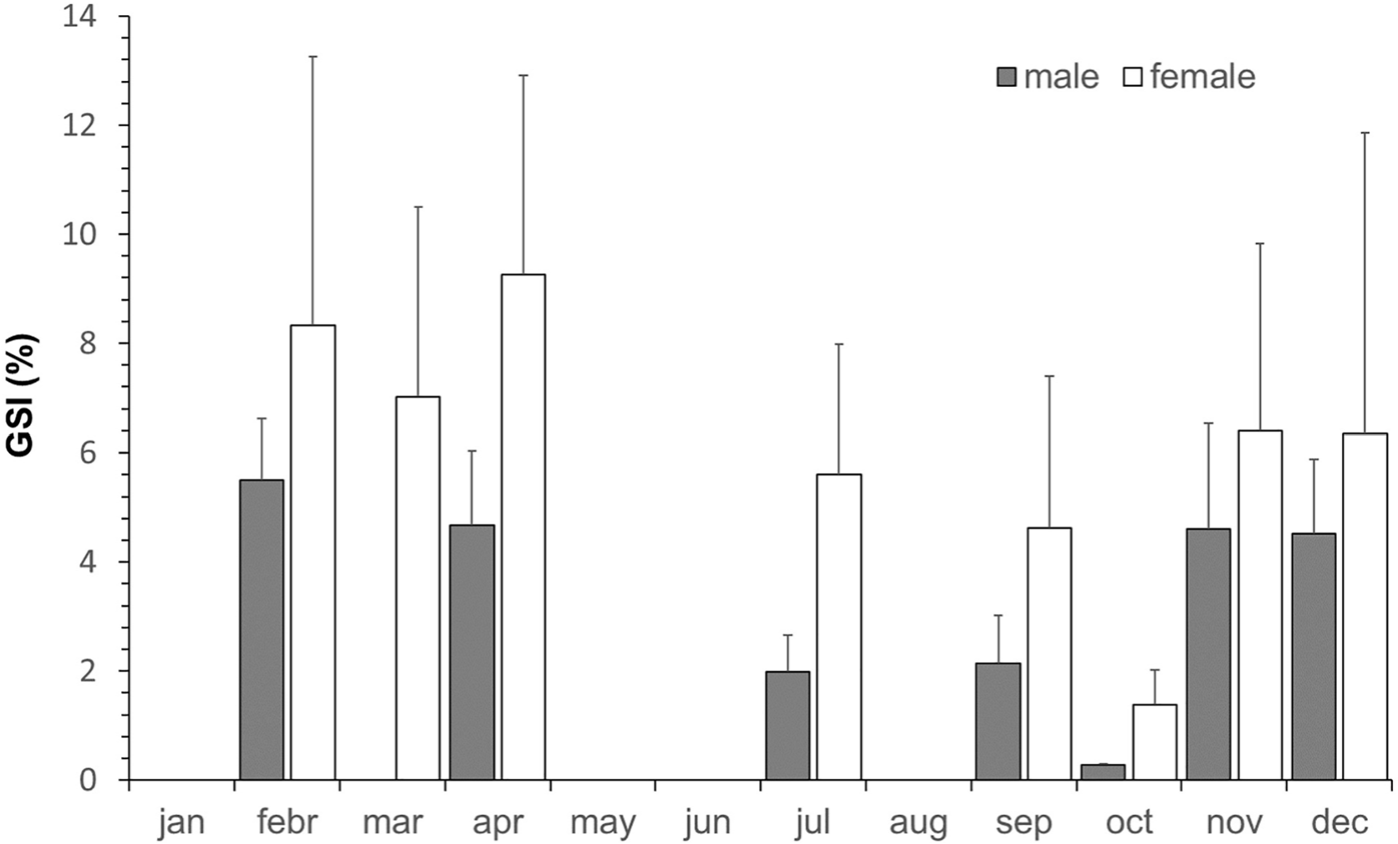
Monthly gonadosomatic index (GSI) for males and females

The reproductive cycle of common carp in Lake Hévíz is outlined in Fig. 3. The high mean value and among individual variance of GSI between February and April indicated the ongoing spawning and the simultaneous representation of individuals that are ready to spawn and had already spawned. During July and September, the ovary of the examined fish contained only eggs with athretized oocytes. From October onwards, oocytes started the vitellogenesis again as it was also indicated by the increasing GSI values. In mature males, sperm-producing individuals were found at all times of the year, except in October.

### In situ induced propagation

The standard body length and the body weight of the smallest female, which was successfully used for in vitro fertilisation was 141 mm and 70 g (Experiment I.). Estimated fertilisation rate in this specimen was about 40%. Induced spawning of common carp in spawning cages (Experiments II. and III.) was also successful. The next day after the hormone treatment at ~ 07.00 h, the spawning substrates in both experiments were covered with fertilised eggs. The estimated fertilisation rate was 70% in Experiment II. and 60% in Experiment III.

## Discussion

According to the literature, the spawning of common carp in temperate regions occurs during spring to first half of summer (mainly in May), when water temperature rises to between 17 and 20 °C (Horváth et al. 2002; Barus et al. 2002; Table 2). At this time, mature, reproductive individuals generally migrate to shallow flooded littorals, selecting areas covered with grass or fine-fibre aquatic vegetation as spawning substrates (characteristic for the phytophilous reproduction guild; Balon 2011). In contrast, in Lake Hévíz, spawning of the dwarf common carp starts from late winter, when the water temperature is characteristically about 27 °C. These observations together with GSI data confirm that oogenesis of females is already complete in late winter, and the fish are in a pre-spawning state. In addition, this population proves that even 1-year-old carp females can produce fertile eggs (Fig. 2d). Horváth (1977, 1985) characterised the relationship between water temperature and maturation time as follows: “If we sum up the days with average temperatures that reach or exceed the lower limit of the spawning temperature (17°C) this value is close to 2500-2700 degree-days during a growing season. For groups that reach maturity in four years, quadrupling this value gives a very good approximation to the values measured for groups kept in warm water. This amount of heat also amounts to 10-11 thousand degree-day …” Applying the same rationale, the sum of degree days in Lake Hévíz for one single season (e.g. from spring 2021 to spring 2022) amounts 11,036 degree-days which explains why common carp could become mature in one year time (Fig. 2). Our observations suggest that males produce sperm for 9–10 months (our observation), while females hold eggs for 12–14 month (present study) after their supposed hatching. The present findings are in accordance with that in the tropical region (i.e. under water temperature range closer to Lake Hévíz) the maturation processes undergo shorter time compared to the temperate zone. For instance, females and males reached sexual maturity at 21.5–22.5 and 15.8–17.5 cm length, respectively, in Ethiopia and Tunisia (Tessema et al. 2020; Hajlaoui et al. 2016), and koi carp weighting 70–200 g were successfully propagated in Bangladesh (Ghosh et al. 2012).

To conclude, females of dwarf common carp of unique thermal Lake Hévíz mature 3 to 4 years earlier and spawn 1 to 3 months before than other carp populations do in the temperate zone.

## Data Availability

The datasets used and/or analysed during the current study are available from the corresponding author on reasonable request.
